# Review on development of potential inhibitors of SARS-CoV-2 main protease (M^Pro^)

**DOI:** 10.1186/s43094-022-00423-7

**Published:** 2022-06-21

**Authors:** Soumya Gulab Katre, Alpana Jagdish Asnani, Kumar Pratyush, Nilima Gangadhar Sakharkar, Ashwini Gajanan Bhope, Kanchan Tekram Sawarkar, Vaibhav Santosh Nimbekar

**Affiliations:** Department of Pharmaceutical Chemistry, Priyadarshini J L College of Pharmacy, Nagpur, MH 440016 India

**Keywords:** SARS-CoV-2 (severe acute respiratory syndrome coronavirus 2), MERS-CoV (Middle East respiratory syndrome coronavirus), M^pro^ inhibitor (main protease inhibitor), Virtual and in vitro screening

## Abstract

**Background:**

The etiological agent for the coronavirus illness outbreak in 2019–2020 is a novel coronavirus known as severe acute respiratory syndrome coronavirus 2 (SARS-CoV-2) (COVID-19), whereas coronavirus disease pandemic of 2019 (COVID-19) has compelled the implementation of novel therapeutic options.

**Main body of the abstract:**

There are currently no targeted therapeutic medicines for this condition, and effective treatment options are quite restricted; however, new therapeutic candidates targeting the viral replication cycle are being investigated. The primary protease of the severe acute respiratory syndrome coronavirus 2 virus is a major target for therapeutic development (M^Pro^). Severe acute respiratory syndrome coronavirus 2, severe acute respiratory syndrome coronavirus, and Middle East respiratory syndrome coronavirus (MERS-CoV) all seem to have a structurally conserved substrate-binding domain that can be used to develop novel protease inhibitors.

**Short conclusion:**

With the recent publication of the X-ray crystal structure of the severe acute respiratory syndrome coronavirus 2 Mm, virtual and in vitro screening investigations to find M^Pro^ inhibitors are fast progressing. The focus of this review is on recent advancements in the quest for small-molecule inhibitors of the severe acute respiratory syndrome coronavirus 2 main protease.

## Background

SARS-CoV-2 (severe acute respiratory syndrome coronavirus 2) is a highly pathogenic beta coronavirus that surfaced in late December 2019 in Wuhan, Hubel Province. SARS-CoV-2 is the seventh human coronavirus (HCV) to be identified, and it is the cause of COVID-19, which was declared a “Public Health Emergency of International Concern” by the World Health Organization (WHO) on January 30, 2020 [1]. COVID-19 symptoms are non-specific and encompass a wide clinical spectrum, making clinical diagnosis without a test difficult. Fever, cough, and anosmia are frequent symptoms; however, many people remain asymptomatic. Asymptomatic patients, as well as those in the symptomatic and pre-symptomatic stages of the disease, can transmit the virus [2].

Many clinical and preclinical researches have been launched to explore feasible treatment options for COVID-19 patients as the number of new cases continues to rise significantly. Many of these possible therapeutic options are based on the repurposing of licensed medications or the evaluation of medications now in clinical trials. As a result, a wealth of information on the pharmacology and toxicity of any potential therapy already exists. In order to assess their efficacy and safety against COVID-19, all available data must be considered in this fast-paced and vital research sector. SARS-CoV-2 is a medium-sized, enveloped, positive-strand RNA virus (30 kb) of the genus Beta coronavirus that appears crown-shaped (corona) in electron micrographs of negatively stained preparations. The viral genome decodes various structural and non-structural proteins that help the virion multiply in a consistent linear pattern during infection.

As a result of M^Pro^ functional role in the viral life cycle, antiviral work against SARS-CoV-2 has proposed viral a viable target [3]. Although the paucity of treatment medicines for SARS-CoV-2 has made disease management problematic for physicians, new research has underlined the importance M^Pro^ of existing medications and their repurposing for illness management. Synergistic use of antimalarial medications like chloroquine–hydroxychloroquine [4] and remdesivir–favipiravir [5], for example, is one of the most well-known recommendations. Huanzhu Lu has also suggested neuraminidase inhibitors, remdesivir, peptide (EK1), abidol, RNA synthesis inhibitors (TDF and 3TC), anti-inflammatory drugs (hormones and other molecules), and Chinese traditional medicine (ShuFengJieDu Capsule and Lianhuaqingwen Capsule) as potential SARS-CoV-2 treatments. The best candidates were 5,7,3′,4′-tetrahydroxy-2′-(3,3 dimethyl allyl) isoflavone, myricitrin, and methyl rosmarinate, which were selected from a library of 32,297 phytochemicals and Chinese medicinal agents with potential antiviral properties against a homology model of the SARS-CoV-2 M^Pro^ (derived from the structures of SARS-COV M^Pro^). Sincalide, ritonavir, phytonadione, and pentagastrin were identified as potential options [6] after testing the activity of FDA-approved medicines against SARS-CoV-2 M^Pro^.

In silico testing of bioactive dietary ingredients against the SARS-CoV-2 M^Pro^ indicated that phycocyanobilin, a chromophore found in cyanobacteria, had a higher binding affinity than nelfinavir, which has been the topic of numerous screenings [7]. Adem et al. also examined 80 flavonoids and discovered that hesperidin and rutin, both present in citrus fruits, had stronger binding affinity than nelfinavir [8]. From a list of antimalarial drugs repurposed for the SARS-CoV-2 M^Pro^, Srivastava et al. discovered that mepacrine, a derivative of chloroquine, had the best in silico results [9]. Salim et al. evaluated a number of chemicals derived from Nigella sativa and discovered that the alkaloid nigellidine was the most effective and discovered that the alkaloid nigellidine and the saponin a-hederin had high binding scores. Sharma et al. found possible M^Pro^ inhibitory action in eucalyptol and jensenone (derived from eucalyptus oil) docking tests.

We devised a system that combines the structures of putative inhibitors, the synthesis process of that drug, virtual drug screening, and in vitro screening to repurpose existing medications to target SARS-CoV-2 M^Pro^, allowing for the quick discovery of antiviral compounds with therapeutic potential [10, 11].

## Main text

### Target therapy

Dai W et al. issued a report in Science during which the two lead molecules 11a and 11b, respectively, developed and manufactured based on the properties of a major SARS-CoV-2 enzyme M^Pro^. Compound 11a, in specifically, is a prospective coronavirus disease 2019 (COVID-19) therapeutic candidate with potential anti infection efficacy, favorable pharmacokinetics, and minimal toxicity [12].

The crystal structure of the SARS-CoV-2 M^Pro^ protein in association with an efficacious inhibitor N3, which was accomplished by the same group, was previously established, creating a crucial basis for this discovery [13]. M^Pro^ is essential to the virus’s life cycle because it can polymerize the protein molecules required for homologous recombination, pp1a and pp1ab, to liberate a sequence of functioning peptides. M^Pro’s^ usefulness in antiviral medication discovery is aided by its conservative ideology in coronavirus and lacking of counterpart in humans [14].

All coronaviruses have evolutionary conserved M^Pro^ receptor subtypes, which frequently include S1′, S1, S2, and S4. As a result, the reasonable design approach can be used to find new SARS-COV-2 inhibitors. The(S)-γ-lactam ring is incorporated to engage with the S1 site since SARS-CoV M^Pro^ inhibitors generally have (S)-γ-lactam ring to inhabit the S1 site. In addition, an aldehyde group is chosen a covalent link with the Cys145 residue’s thiol. Because the S2 region may hold a big group, a cyclohexyl or 3-fluorophenyl group with a broad geographical volume is added at the appropriate location. After that, an indole group is added to the S4 domain in order to boost drug-like characteristics by forming intermolecular hydrogen bonds. Finally, a method for obtaining the lead compounds 11a and 11b is invented [15].

**Fig. 1:**
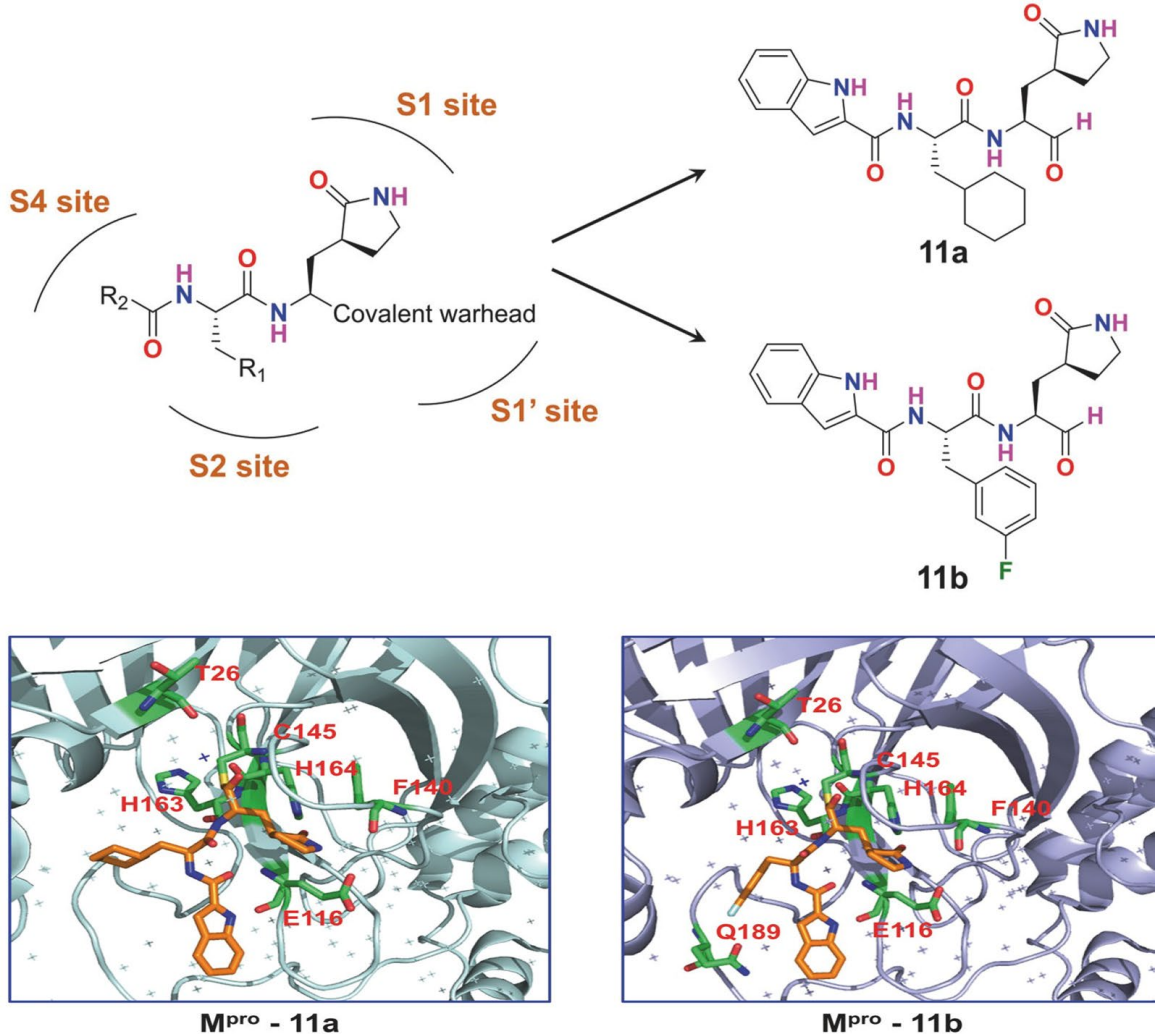
Discovery of drug targeting M^pro^ against COVID-19

The crystal structures of M^Pro^ in association with 11a (PDB code: 6LZE) and 11b (PDB code: 6M0K) have been revealed at a resolution of 1.5 Å. to understand the antagonistic mechanism of 11a and 11b. 11a and 11b follow a common antagonistic binding mechanism, as seen by the structures. In the S1′ area, the aldehyde group is making a covalent link with Cys145, while the (S)-γ-lactam ring and the indole group establish hydrophobic interaction bonds with the S1 and S4 areas, appropriately. The stereo-structure and electron density variations between cyclohexyl and 3-fluorophenyl groups were most likely to blame for a little discrepancy between 11a and 11b in the S2 domain. Numerous hydrogen atoms, in particular, played a role in the hydrophobic interactions of protein–ligand complexes. Altogether, the interacting mechanisms of compounds 11a and 11b mostly with M^Pro^ are reminiscent to those of compounds N1, N3, and N9, which have been documented as broad-spectrum antagonists of the coronavirus M^Pro^ (Fig. 1).

### SARS-M^PRO^ crystal structures

**Fig. 2:**
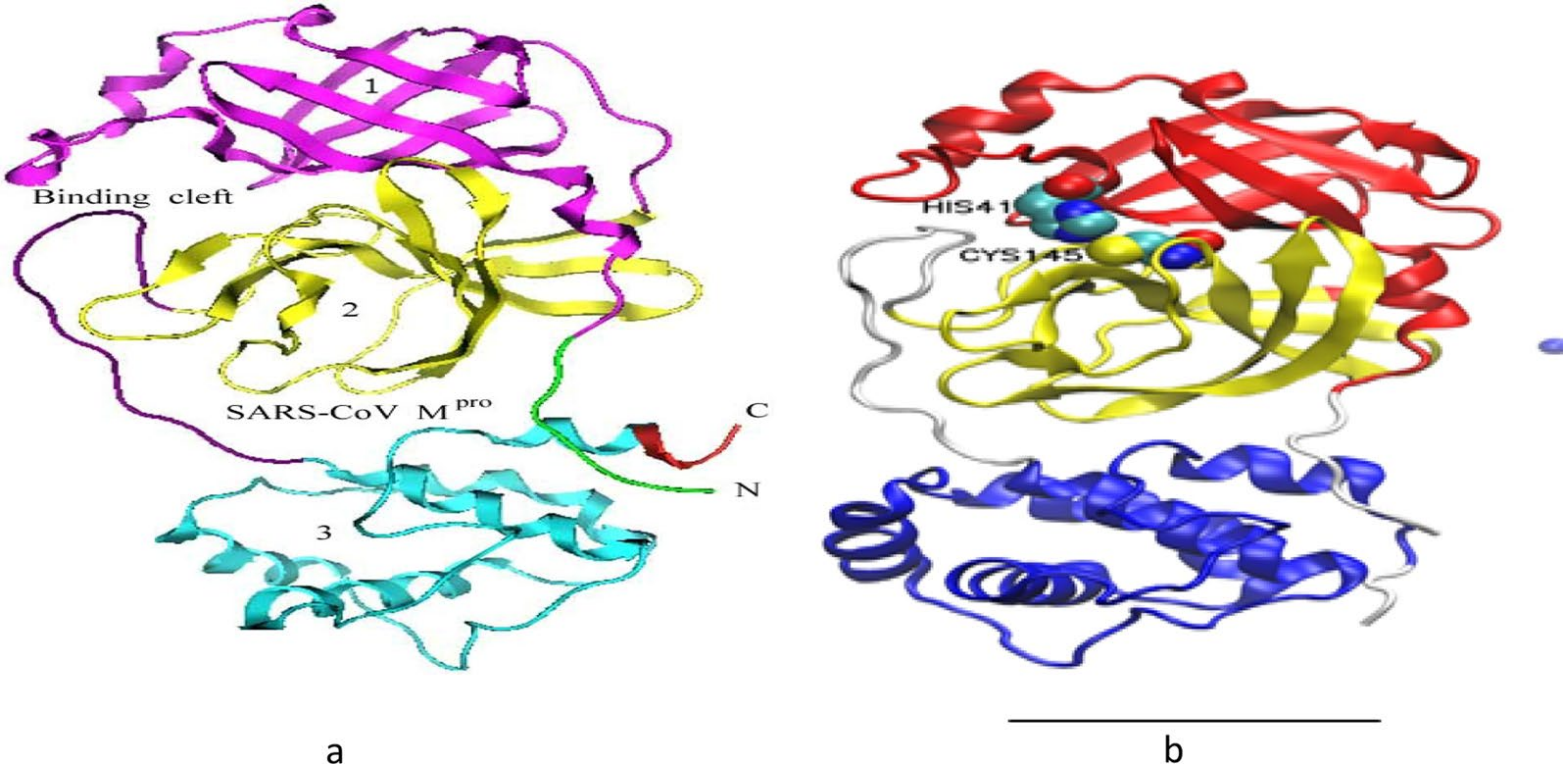
**a** The ribbon representation of the crystal structure of the SARS-CoV-2 M^pro^ from PDBID: 6Y2E. Domains I, II and III are displayed in pink, yellow and light blue (teal) respectively. The connection region between II and III are in red (C), green (N) and violet (binding cleft). **b** The ribbon representation of the crystal structure of the SARS-CoV-2 M^pro^ from PDBID: 6Y2F. Domains I, II and III are displayed in red, yellow and blue respectively. The connection region between II and III is in white and the catalytic dyad residues (His41 and Cys145) are in solid sp

M^Pro^, the primary protease of the coronavirus, is a cysteine protease with a 2 different structure (domains I and II) associated with a C-terminal-helical domain III. Domains I and II have a structure that is comparable to that of chymotrypsin-like serine protease. M^Pro^, the primary protease of coronaviruses, is a cysteine protease with such a 2 different structure (domains I and II) connected together (Fig. 2a) [16, 17].

304 residues compensate the enzyme molecule. As can be seen in picture, the complete peptide chain is folding into three domains: domain 1, domain 2, and domain 3. The N-terminal (res. 1–7) is characterized by the letter N, whereas the C-terminal is signified by the letter C. The binding cleavage is at the junction of domains 1 and 2.

In early 2020, the SARS-CoV-2 M^Pro^ crystal estimation was done to 2.1 A resolution in conjunction with a chosen mechanistic inhibitor (ligand N3; Fig. 2b). The structure of M^Pro^ with the ligand N3 highlighted a number of important aspects of inhibitor–protein interactions. The updated version of the ligand N3 in the M^Pro^ binding pocket yielded a plethora of information on the role of various residues. In the presence of this inhibitor, hydrogen bonding (residuesPhe140-A, Gly143-A, His163-A,His164-A,Glu166-A,Gln189-AandThr190-A) and hydrophobic interactions (residues His41-A, Met49-A, Tyr54-A, Met165-AandLeu167-A) work together to settle the molecule deep inside the M^Pro^ active site, thereby anchoring it in place [18].

We go over the precise interactions of N3 with M^Pro^ in this section (Fig. 1c, d). The electron density reveals that the S atom of protomer A establishes a covalent connection (1.8) with the C atom of the vinyl group, confirming the Michael addition. Gln at the P1 location is an essential requirement for the S1 subsite. The S1 subsite is produced of the side chains of F140, N142, E166, H163, and H172 of protomer A, and S1 of protomer B—as well as the backbone chains of F140 and L141 of protomer A—and two ordered intermolecular forces (which we refer to as W1 and W2). P1’s lactam inserts into the S1 subsite, forming a hydrogen connection with protomer A’s H163. The side chain of Leu at the P2 site penetrates significantly into the hydrophobic S2 subsite, which encompasses the side chains of H41, M49, Y54, and M165, and also the alkyl part of D187’s side chain. Val at P3 has a hydrocarbon side chain, indicating that this site can tolerate a variety of functional groups [19].

The side chains of M165, L167, F185, Q192 of protomer A, as well as the main chain of Q189 of protomer A, envelope the side chain of Ala on the P4 side, establishing a tiny hydrophobic pocket. P5 has dipole–dipole interactions with protomer A’s P168 and the strand of residues 190–191.

### Role of N3 and N1

**Fig. 3:**
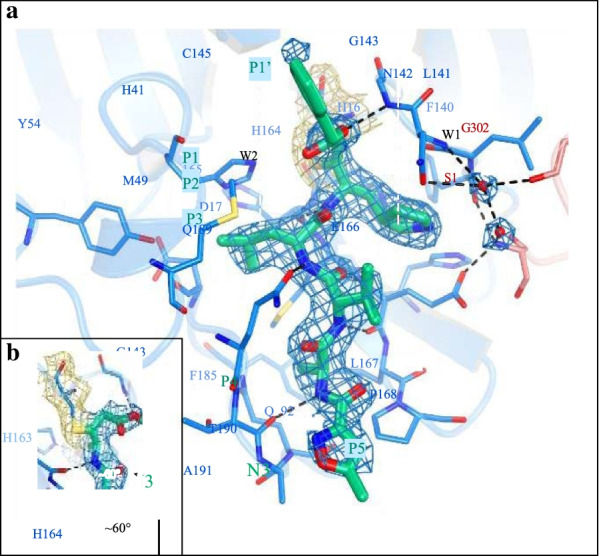
**a** The substrate-binding pocket in considerable detail. The important residues that help compensate the binding pocket are represented by sticks, while the two hydrogen bonds (W1 and W2) are represented by circles spheres of red the P1, P1′, P2, P3, P4, and P5 N3 sites are shown. Black dashed lines signify hydrogen bonding that enable to lock the inhibitor. Across the N3 molecule (blue mesh), C145 of protomer A (yellow mesh), and the two liquids, the 2Fo Fc density map scaled at 1.2 is illustrated (blue mesh). **b** The C-S covalent bond

The Michael addition of the protease’s catalytic Cys145 to inhibitor N3 usually causes suppression of the SARS-CoV-2 M^Pro^, comparable to inhibitor N1 (Fig. 3a) with the SARS-CoV M^Pro^ [20]. Inside a two-step irreversibly suppression process, the enzyme inhibition occurred in a time-dependent manner. The inhibitor develops a non-covalent link well with enzyme before building a stable covalent bond. Numerous hydrophobic, van der Walls, and hydrogen-bonded engagements maintain the inhibiting molecule only within substrate-binding region, according to molecular docking. The dissociation constant Ki and the inactivation rate constant k3 for covalent binding interactions could not be quantified because antagonist was effective and the inactivation of the enzyme was quick.

**Fig. 4:**
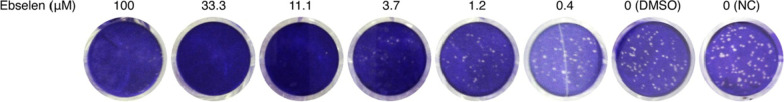
Covalently bound inhibitors used for the SARS-CoV-2M^pro^ (N3) and the SARS-CoV M^pro^ (N1)

The Michael acceptor has a pseudo-second-order deactivation constant of 11,300 T 800 M—1 s—1, implying a minimal dissociation constant and quick covalent deactivation by the Michael acceptor, which is important in minimizing cross-reactivity with other enzymes and pharmacological side effects. It exhibited a CC50 value of greater than 133 mM. Covalent bonding to Cys145 became a significant criterion for the identification of antagonists because of the resemblance in interactions between N1 and the SARS-CoV M^Pro^ (Fig. 4).

### Antiviral activity assay

**Fig. 5:**
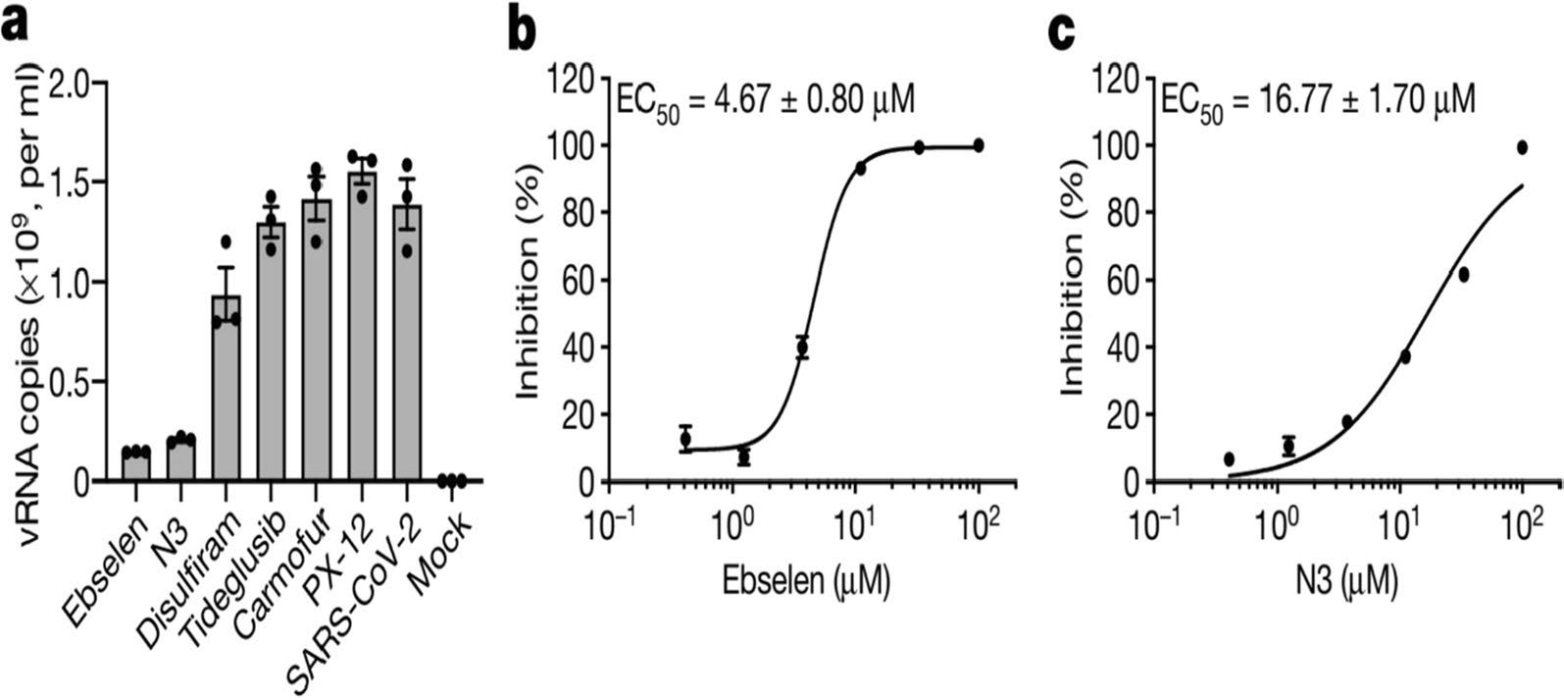
Images from the ebselen plaque-reduction assay. With respect to the negative control (NC) and the positive control (PC), the number of plaques decreases as the concentration of ebselen increases DMSO. The results are a composite of four biological replicates. The drug’s antimicrobial properties against SARS-CoV-2. **a** qRT-PCR analysis was used to estimate the number of absolute viral RNA (vRNA) copies (per ml) in the supernatant 72 h after infection. The data represent the mean s.e.m. of three biological replicates, ebselen dose–response curves, and **b** N3, **c** plaque-reduction assay. All data are presented as mean s.e.m., based on four biological replicates

The test began by mixing 0.2 µM SARS-CoV-2 M^Pro^ various substrate concentrations (2.5–100 µM) right away. An Visualize wideband screen reader was used to measure fluorescence intensity (PerkinElmer). The linear part of the curves was fitted to a straight line to obtain the changes are taking place. A double-reciprocal plot was used to derive the thermodynamic properties Km and kcat. Because N3 is an irrevocable mechanism-based antagonist for SARS-CoV-2 M^Pro^, kobs/[I] was employed as an estimate of the pseudo-second-order rate constant to assess the inhibitor’s suppressive activities. In this scenario, 0.2 µM enzyme, 20 µM substrate, and 6 different doses (0–1 µM) of inhibitor were used in the experiment. We tested if these drugs might block viral replication in cell-based assays to back up the enzymatic inhibition results in vitro. As shown in Fig. 5, in SARS-CoV-2-infected Vero cells, quantitative real-time RT-PCR (qRT-PCR) demonstrated that ebselen and N3 had the strongest antimicrobial activity amongst those compounds at a dose of 10 µM pretreatment. Further to evaluate the efficiency of these two drugs in making antibodies, we conducted a plaque-reduction experiment (Extended Data Fig. 5).

**Fig. 6:**
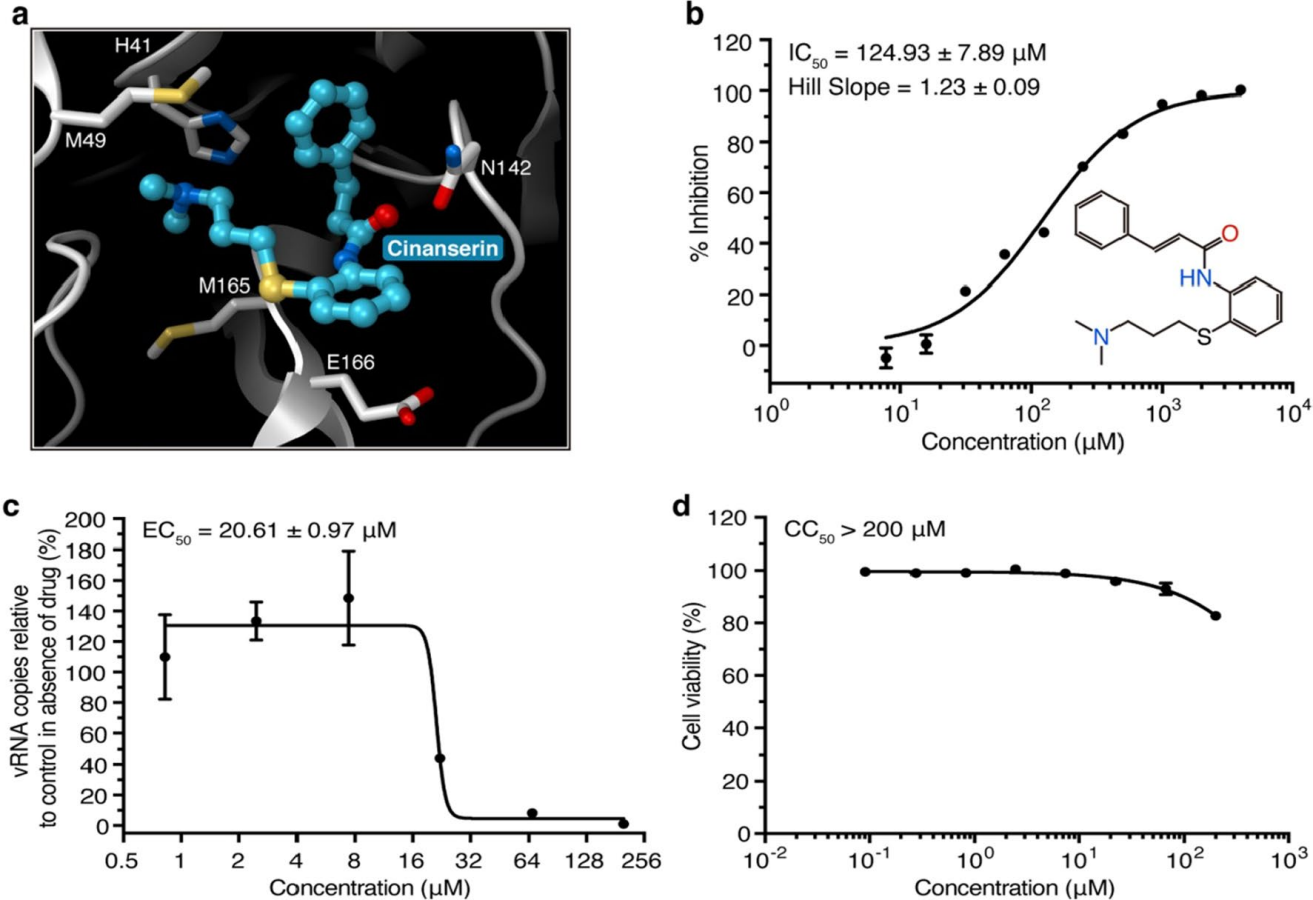
**a** Cinanserin’s docking performance. SARS-CoV-2 M^Pro^’s structure is depicted as a white cartoon, with cinanserin depicted as cyan balls and sticks, and residues anticipated to interact with cinanserin depicted as sticks. **b** Cinanserin’s suppressive action on M^Pro^. **c** qRT-PCR analysis of cinanserin’s antiviral activity. **d** Cinanserin cytotoxic activity test in Vero E6 cells. All data are presented as mean s.e.m., based on three biological replicates

With half-maximal appropriate database (EC50) of 4.67 M and 16.77 M, respectively, ebselen and N3 inhibited SARS-CoV-2 (Fig. 6b, c). Both of these molecules’ dose–response curves show that they will be able to breach the cell wall and reach their destinations. Ebselen is an anti-inflammatory, anti-oxidant, and cytoprotective organo-selenium molecule. This substance has been studied in the past for the treatment of a variety of ailments, including bipolar illness and cognitive impairment [21].

#### Extended data

Ebselen does have low cytotoxicity (the median lethal dose in rats when given orally is > 4,600 mg kg1), and its safety in humans has also been tested in several clinical trials [22]. These findings appear to suggest that ebselen could be used to treat coronaviruses in the clinic. In addition, qRT-PCR research revealed that cinanserin had a modest inhibitory effect against SARS-CoV-2, with an EC50 value of 20.61 M (Extended Data Fig. 6). This number is higher than that of the enzymatic inhibition testing, implying that cinanserin could be a multifunctional target in the prophylaxis of viral infection. Afterward, drug-resistant variants will be generated and analyzed in order to better understand the method of action of cinanserin.

### Viral protease enzyme

M^Pro^, the viral primary 3-chymotrypsin-like cysteine protease, has been identified as an important drug discovery target for SARS-CoV-2. M^Pro^ is known to govern coronavirus replication and is required for viral life cycle. Domain I (Phe8-Tyr101), domain II (Lys102-Pro184), and domain III (Thr201-Val303) were related by a loop of residues Phe185 to Ile200 in the viral protease. In the split between domains I and II of SARS-CoV-2 M^Pro^, the active pocket with catalytic dyad (Cys145 and His41) was defined.

The replication of SARS-CoV-2 is mediated by a complex made up of two polyproteins that are translated from viral RNA. The catalytic residues in M^Pro^ cleave these polyproteins in at least 11 places around the C-terminal and central regions, releasing the essential proteins for viral replication [23]. SARS-M^Pro^ CoV-2 is divided into three domains (Fig. 2a): domain (residues 8–101), domain (residues 102–184), and domain (residues 201–303). The first two domains have an antiparallel b-barrel structure, but the third domain (residues 185–200) forms an antiparallel conglomerate with five a-helices, which is linked to the first two by a lengthy loop region. The M^Pro^ of SARS-CoV viruses has a Cys-His catalytic dyad, with the substrate-binding site sandwiched between domains, and CoV M^Pro^ has a structurally highly conserved substrate-recognition pocket, which makes them an attractive target for drug design and development. The recent finding of novel CoVs, as well as structural data on CoV M^Pro^ from various strains, has opened up new avenues for research. The superposition of 12 M^Pro^ crystal structures (SARS-CoV-2, SARS-CoV, MERS-CoV, HCoV-HKU1, BtCoV-HKU4, MHV-A59, PEDV, FIPV, 312 TGEV, HCoV-NL63, HCoV-229E, and IBV) [24–32] indicated that all CoV M^Pro^ has the same substrate-binding area between domains as a result.

The crystal structure of the SARS-CoV-2 M^Pro^ showed Michael addition of the Sg-atom of the catalytic Cys145 to the pi-bond of the unsaturated ester group in the mechanistic inhibitor N1 (Fig. 3a), and a water molecule stabilized the inhibitor by hydrogen bonds to the carboxyl a. To inhibit Cys145 of the SARS-CoV M^Pro^, Zhu et al. [33] utilized highly electrophilic peptidomimetic aldehydes as warheads, whereas Zhang et al. [34] employed a-ketoamide and Michael acceptor-based hybrid inhibitors. A review by Hilgenfeld [35] provides a detailed explanation of structure-based medication development for previous CoV M^Pro^. Other forms of peptidic and peptidomimetic inhibitors with different electrophilic functional groups, such as halomethyl ketones, epoxyketones, nitriles, and phthalhydrazide ketones, were synthesized to establish covalent binding of inhibitors to the catalytic cysteine of the SARS-CoV M^Pro^. In cell culture, all of these drugs successfully suppressed SARS-CoV multiplication. The SARS-CoV and SARS-CoV-2 M^Pro^ have a conserved active site domain, which should allow inhibitors of the former to target the latter.

### Mechanism of Mpro inhibitors

It is worth noting that the SARS-CoV-2 M^Pro^-N3 inhibitor complex exhibited intermolecular molecular contact via the creation of four hydrogen bonds with Cys145, Glu166, and Gln189, as well as additional intermolecular interactions. R428 and UK-432097 in the active pocket of viral protease displayed a maximum of four hydrogen bonds in SARS-CoV-2 M^Pro^ FDA-authorized medication complexes. Furthermore, both FDA medicines and the N3 inhibitor demonstrated significant hydrophobic, polar, negative, positive, and glycine interactions with common residues in the SARS-CoV-2 M^Pro^ active pocket.

### Study parameters

#### Virtual screening

The use of high-performance computer to screen large-small molecule databases for possible ligands against a specific therapeutic target is known as virtual screening [36, 37]. Following that, the top 10 docked medicines were chosen for re-docking analysis in AutoDock Vina, which exhibited better binding scores and occupied the same location in the protease active pocket as shown in [38–40].

#### Re-docking and free binding energy calculation

Following that, the top 10 docked poses of protein–drug complexes from virtual screening were recovered and re-docked. Finally, utilizing the ligand–receptor interaction module, the top poses with the greatest docking score and lowest RMSD were chosen for intermolecular interaction profiling [41]. Under default settings, numerous intermolecular interactions between ligands and active residues of proteins, such as hydrogen bonding, hydrophobic, p-cation, p–p contact, contact, negative, positive, glycine, polar, and salt bridge formation, were estimated. As previously reported, the pocket encompassing the same active residues was created using the Chimera1.14-AutoDock Vina plugin configuration [42, 43].

#### Molecular dynamics simulation analysis

MD simulation is a widely established computer approach in drug development for understanding physical interactions at the atomic level for biological macromolecules, such as structure–function connections, intramolecular/intermolecular interactions, and other structural characteristics.

#### Post-molecular dynamics

Snapshots from corresponding MD simulations were evaluated for binding affinity using the Prime MM/GBSA technique to calculate the effect of MD simulation on the binding free energy of ligands with the active pocket of SARS-CoV-2 M^Pro^. The total DGBind and individual energy component values were estimated on snapshots from simulated trajectories. There was no significant change in the net binding energy for any of the SARS-CoV-2 M^Pro^ FDA-authorized medicines in any of the snapshots [39, 44–48].

## Conclusions

The SARS-CoV-2 epidemic has spurred scientists from many walks of life to contribute to the rapid development of possible cures or vaccinations. The active site of the newly discovered SARS-CoV-2 M^Pro^ appears to be highly versatile, according to the findings of this virtual screening and molecular modeling investigation. The characteristics of a key SARS-CoV-2 enzyme M^Pro^ were used to design and synthesize lead compounds which is a potential coronavirus disease 2019 (COVID-19) treatment candidate in particular. M^Pro^, the coronavirus’s major protease, is a cysteine protease with two distinct structures (domains I and II). The SARS-CoV-2 M^Pro^ crystal estimate was performed in early 2020 in combination with a selected mechanistic inhibitor. SARS-CoV-2 M^Pro^ suppression is generally caused by Michael’s Cys145 and N3. Before forming a stable covalent connection, the inhibitor forms a non-covalent bond with the enzyme. According to molecular docking, inhibition occurs in a time-dependent way. M^Pro^, a 3-chymotrypsin-like cysteine protease found in SARS-CoV-2, has been identified as an interesting drug discovery target. M^Pro^ is essential for the viral life cycle and is known to regulate coronavirus replication.

## Abbreviations

SARS: Severe acute respirator syndrome
MERS: Middle East respiratory syndrome
CoV-2: Coronavirus 2
M^Pro^ inhibitor: Main protease inhibitor
vRNA: Viral ribonucleic acid
NC: Negative control
PC: Positive control
WHO: World Health Organization
FDA: Food & drug administration
